# Dramatic Response of a Large, 10 Cm Hepatocellular Carcinoma to Monotherapy with Yttrium-90 Based Selective Internal Radiation Therapy

**DOI:** 10.7759/cureus.425

**Published:** 2015-12-22

**Authors:** Tejan Diwanji, Tuo Dong, Fred Moeslein, Michael Chuong

**Affiliations:** 1 Radiation Oncology, University of Maryland Medical Center; 2 College of Medicine, Howard University; 3 Interventional Radiology, University of Maryland Medical Center

**Keywords:** sirt, tumor embolization, radioactive embolization, hepatocellular carcinoma

## Abstract

Hepatocellular carcinoma (HCC) is predominantly diagnosed in advanced stages and not amenable to surgical resection and transplantation. Systemic therapies have had a limited efficacy in treating HCC. Although HCC is a radiosensitive tumor, treatments with external-beam radiation are limited by radiosensitivity of normal liver tissue and surrounding organs-at-risk, i.e. bowel, stomach, and kidney. Several large retrospective series have demonstrated a modest effect of selective internal radiation therapy (SIRT) with Yttrium-90 (^90^Y) microspheres in unresectable HCC, both in terms of tumor response and survival. The authors present a patient with an extremely large, multifocal, unresectable HCC who achieved a dramatic response with SIRT treatment.

## Introduction

Hepatocellular carcinoma (HCC) is the fifth most commonly diagnosed cancer in men, and seventh among women, worldwide [1]. HCC is also the most common primary malignancy of the liver and is the second leading cause of cancer-related death in the world [1]. Early stages of HCC can be treated with curative resection and liver transplantation, but fewer than 40% of the patients are eligible for such a treatment. The majority of patients present with unresectable disease, with local treatment options being limited to transarterial chemoembolization (TACE) or selective internal radiation therapy (SIRT). In recent years, there is a growing body of evidence supporting the therapeutic efficacy and safety of Yttrium 90 (^90^Y)-based SIRT in the treatment of advanced stages of HCC [2-4]. 

## Case presentation

The patient was consented and enrolled on an institutional research protocol prior to treatment. The University of Maryland Medical Center  Institutional Review Board approved the study (protocol #GCC 0346).

JA is a 57-year-old Caucasian female who presented with right-sided abdominal pain, associated nausea, and fatigue. She had a past medical history of thalassemia, requiring plasmapheresis and eventually splenectomy. She had no history of hepatitis or alcohol abuse. On physical examination, she appeared fatigued and exhibited mild right upper quadrant tenderness without jaundice. Her liver was palpable 3 cm below the right costal margin without abdominal distension.

**Figure 1:**
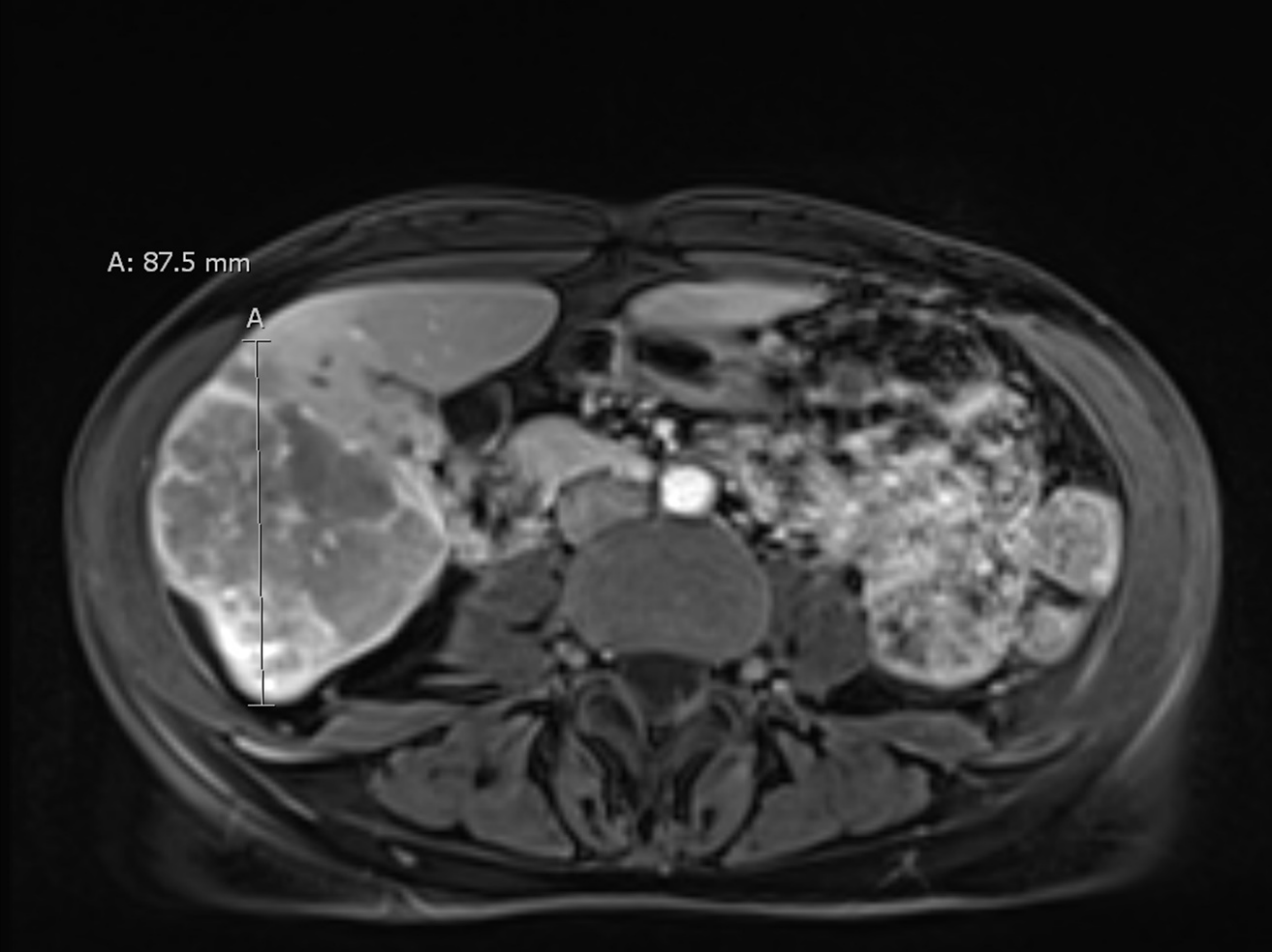
Representative axial slice from pre-treatment MRI

**Figure 2:**
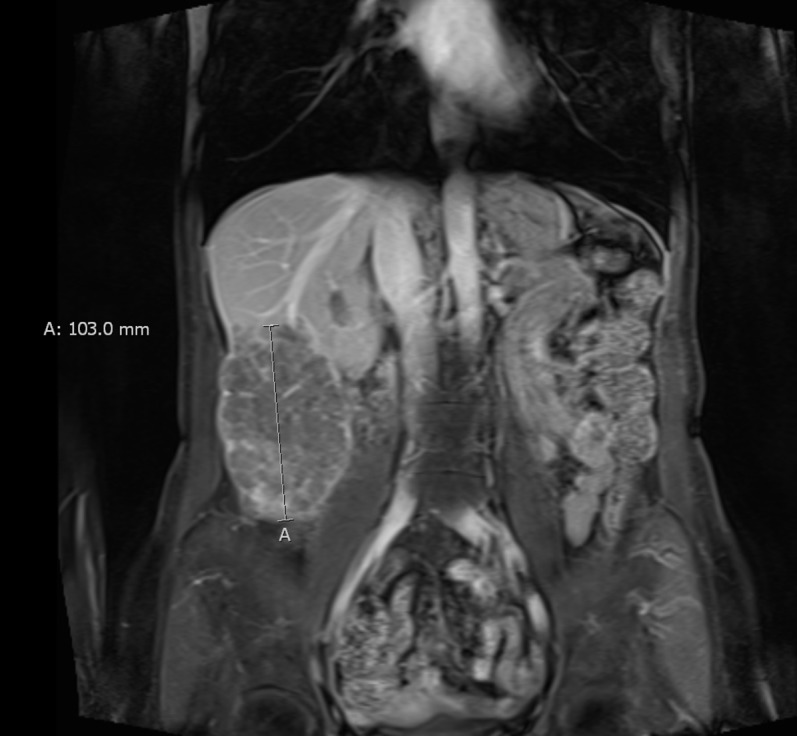
Representative coronal slice from pre-treatment MRI

An MRI of the abdomen demonstrated two large T2 hyperintense masses in the right hepatic lobe, specifically segments six and seven of the liver, which measured 10.3 x 8.8 cm and 1.2 x 1.2 cm, respectively (Figures 1-2). Tumor extension into the right-sided branches of the portal vein was also noted. No evidence of abdominal or pelvic metastasis was seen on MRI. At the time of initial presentation, her liver function was well-preserved; her Childs-Pugh score was A5. Her AFP was 9,260 ng/ml. The constellation of findings was consistent with a diagnosis of HCC.

The patient’s case was reviewed in a multidisciplinary conference and the recommendation was to treat with sorafenib and SIRT with ^90^Y Sir-Spheres (Sirtex SIR-Spheres Pty Ltd, Australia). When presented with the recommendations, however, the patient elected to proceed with SIRT alone due to her concerns regarding exacerbation of fatigue with sorafenib. 

She underwent a Technetium-99m microaggregated albumin (MAA) scan to assess the hepatic arterial vasculature and quantify the pulmonary shunting, which is routinely performed prior to SIRT. The hepatic-pulmonary shunt was calculated to be 1.41%, within our institutional guidelines of <20%. An angiogram was performed to evaluate the right-sided tumor burden and was estimated at 35%. Total liver, right hepatic lobe, and left hepatic lobe volumes were estimated using the patient’s pre-treatment MRI and were 1,597 cc, 1,091 cc, and 506 cc, respectively. Dose calculations were adapted from the formula recommended in the Sirtex SIR-Spheres Pty Ltd manual with a minor alteration to account for the body surface area. One week after her MAA scan, JA underwent her SIRT procedure under fluoroscopic guidance with a prescription dose of 34.59 mCi. Two weeks later, she presented to the emergency department with right upper quadrant and epigastric abdominal pain, which resolved with symptomatic treatment.

**Figure 3:**
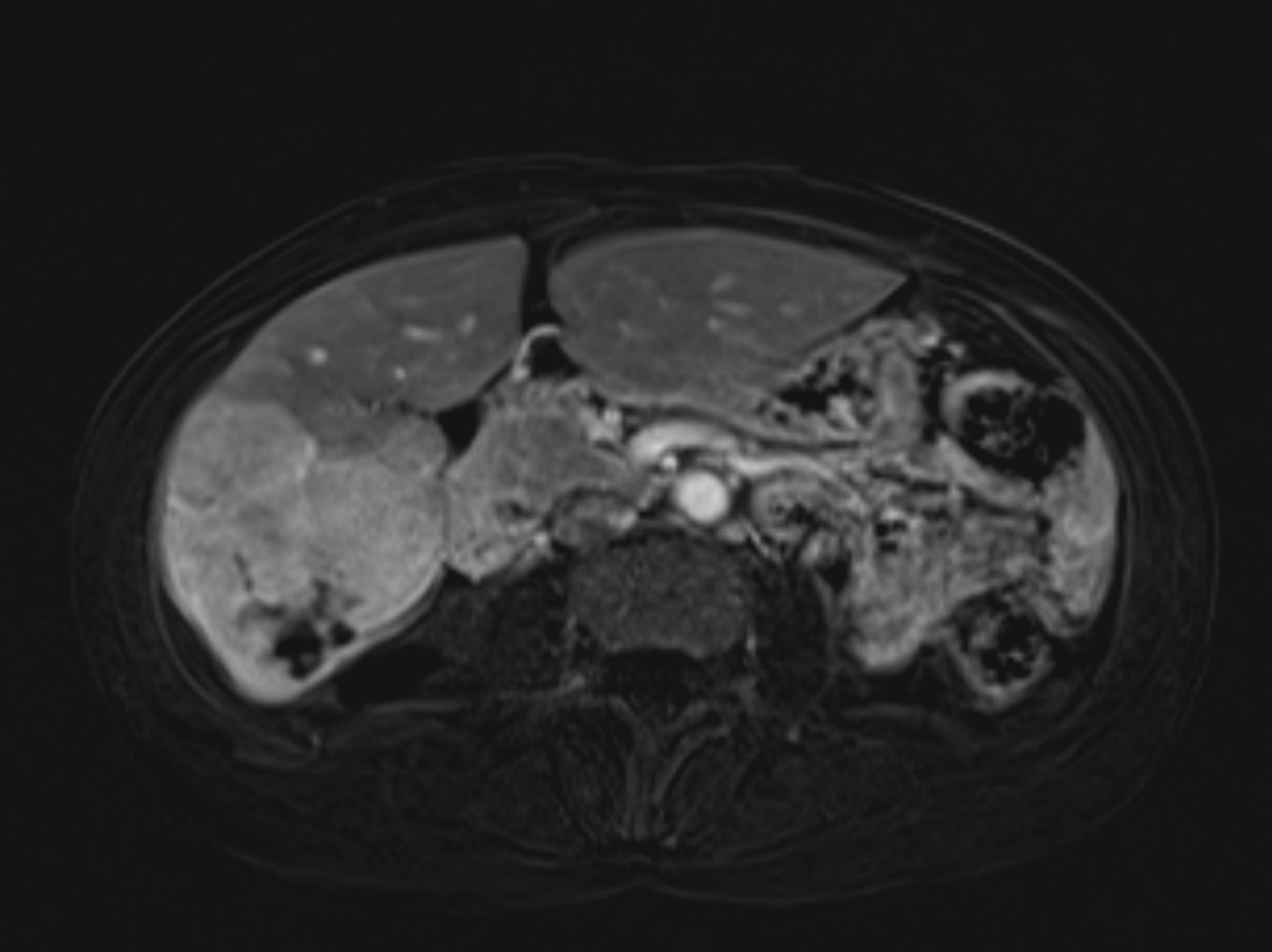
Representative axial slice from MRI obtained after first SIRT procedure

**Figure 4:**
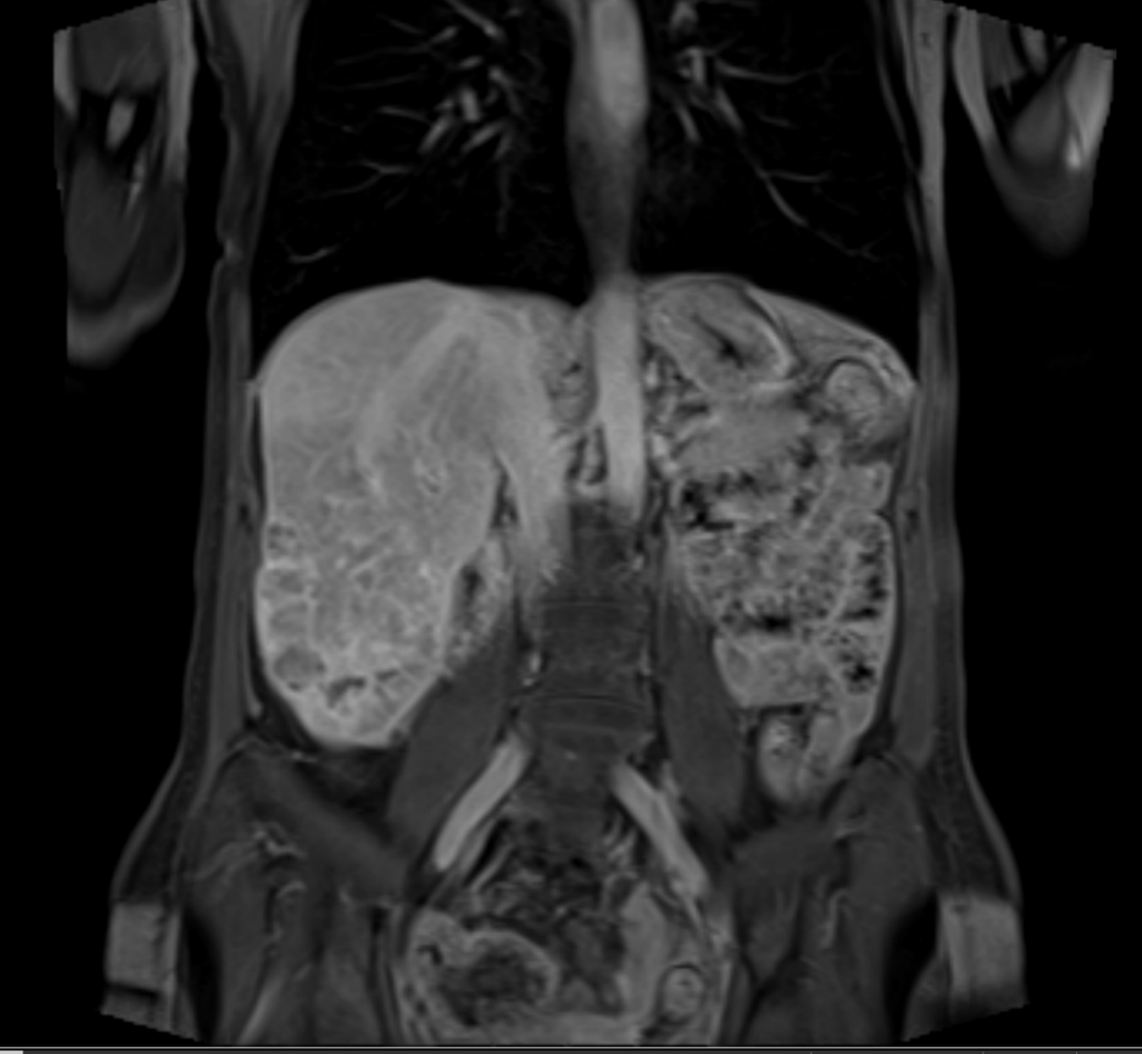
Representative coronal slice from MRI obtained after first SIRT procedure

Follow-up MRI of the liver obtained five weeks after treatment showed the dominant mass located in segment six was stable in size with small areas of necrosis and increased arterial enhancement throughout, which suggested residual tumor (Figures 3-4). The satellite lesion in segment seven remained stable, compatible with persistent tumor as well. The presence of persistent disease, in the setting of deferred systemic therapy, prompted a discussion of retreatment with SIRT. Although uncommon, retreatment with SIRT was considered due to the large tumor burden in her right lobe and favorable post-treatment liver function. The patient was counseled on the potential for increased side effects in the re-treatment setting. She underwent a repeat SIRT procedure, eight weeks after her initial treatment with a dose of 28.1 mCi administered to the right hepatic artery territory under fluoroscopic guidance. She tolerated the procedure well and was discharged home the same day.

**Figure 5:**
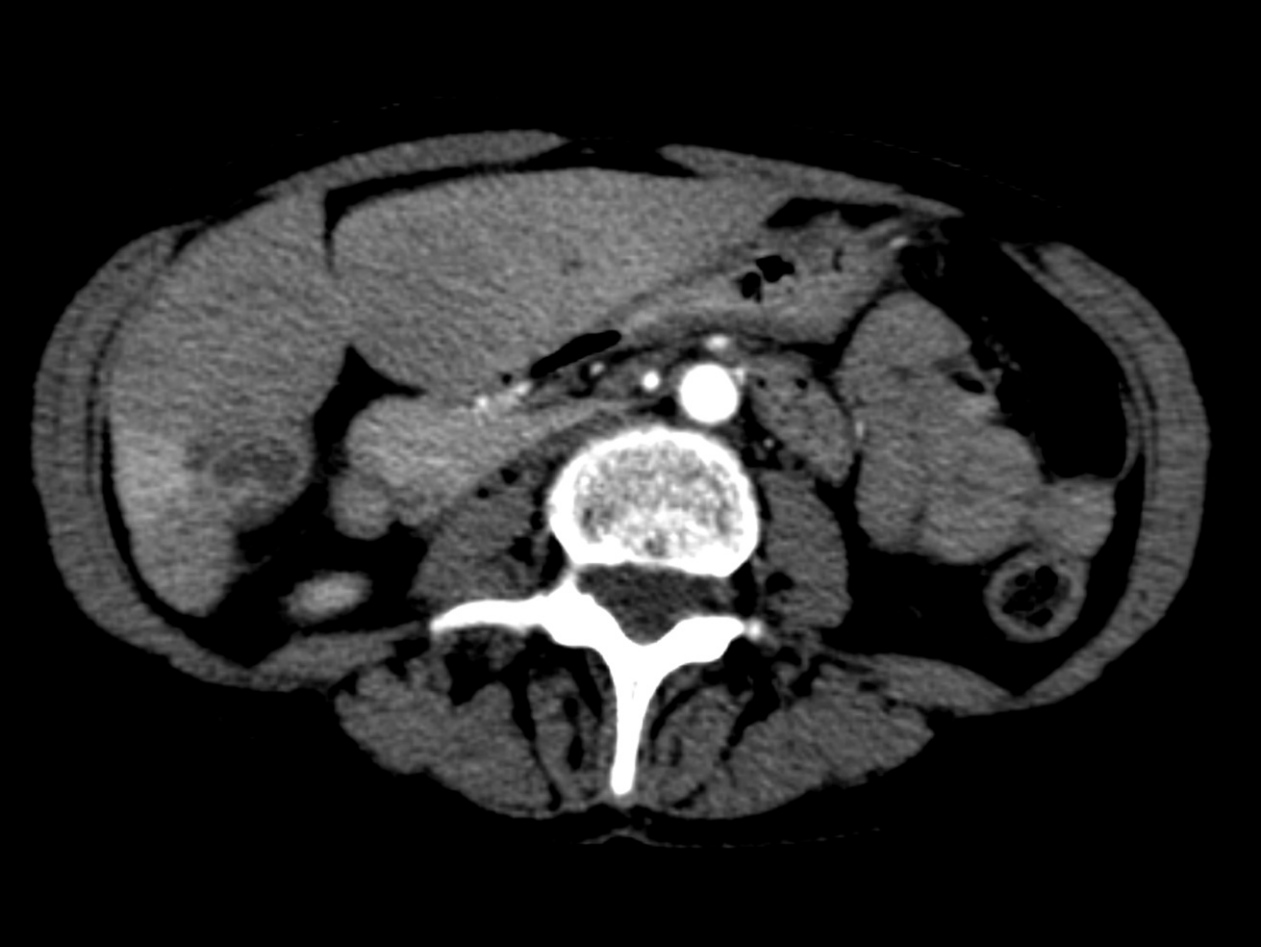
Representative axial slice from CT obtained three months after second SIRT procedure

**Figure 6:**
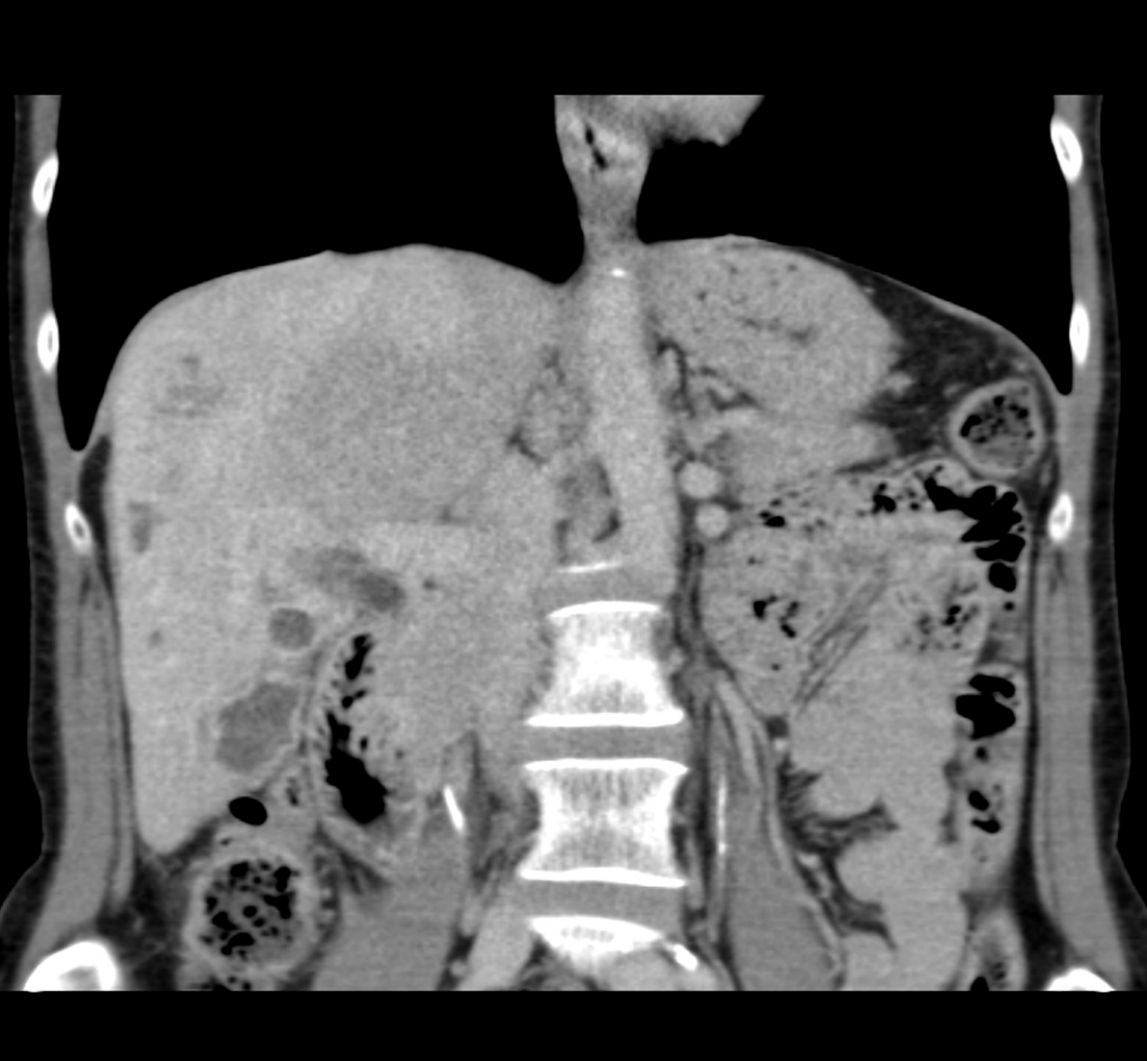
Representative coronal slice from CT obtained three months after second SIRT procedure

**Figure 7:**
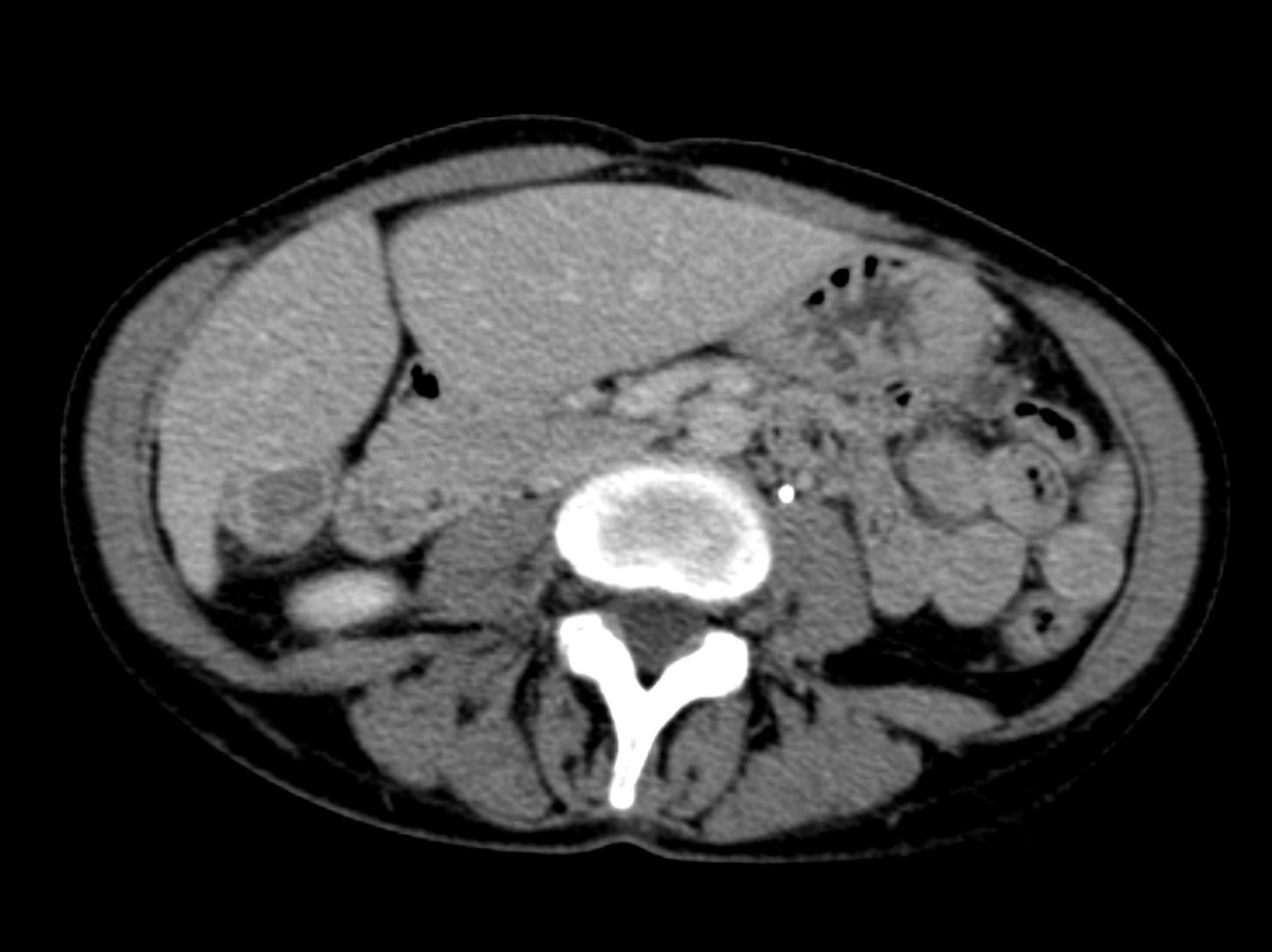
Representative axial slice from CT obtained six months after second SIRT procedure

**Figure 8:**
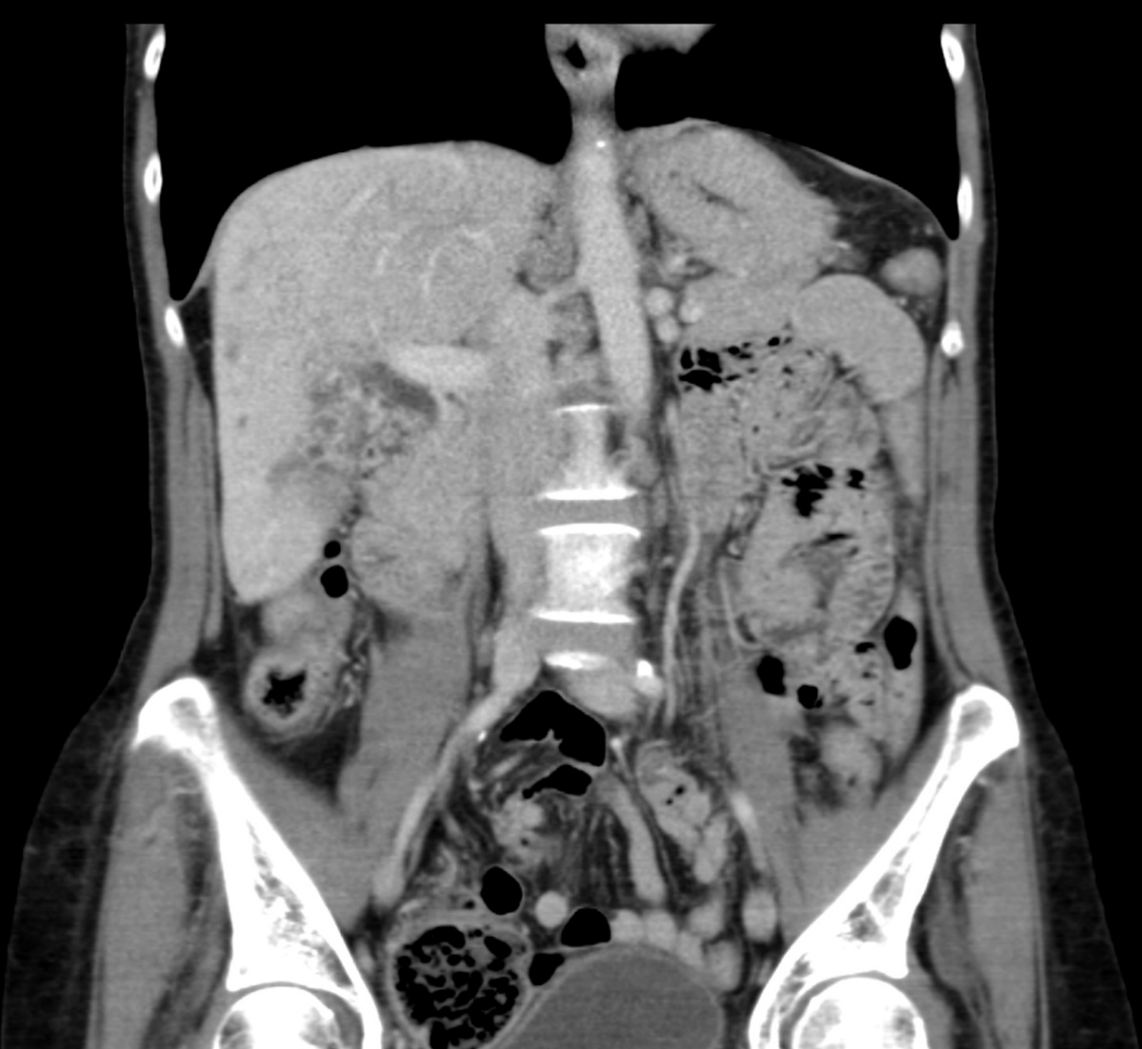
Representative coronal slice from CT obtained six months after second SIRT procedure

**Figure 9:**
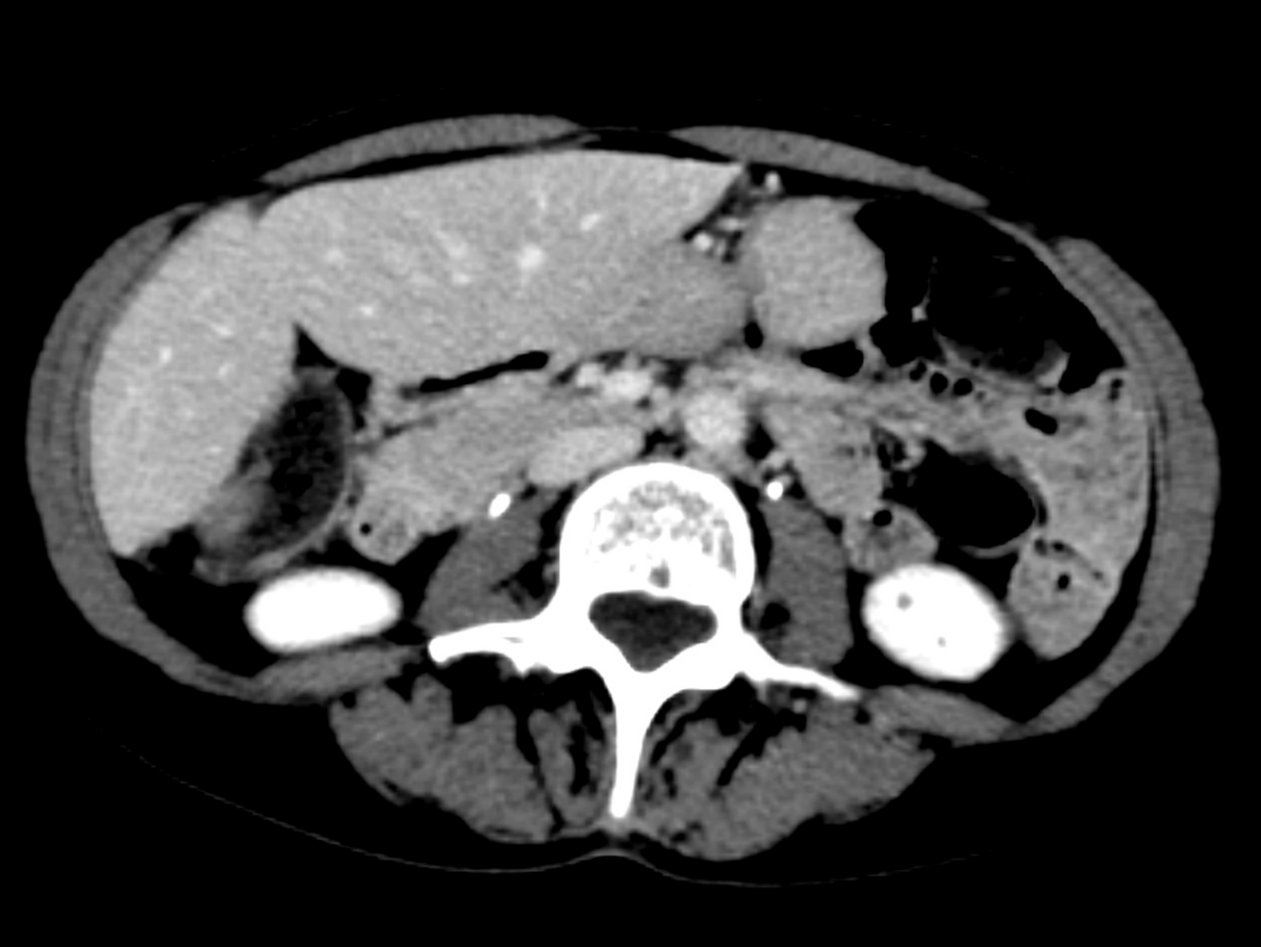
Representative axial slice from CT obtained nine months after second SIRT procedure

**Figure 10:**
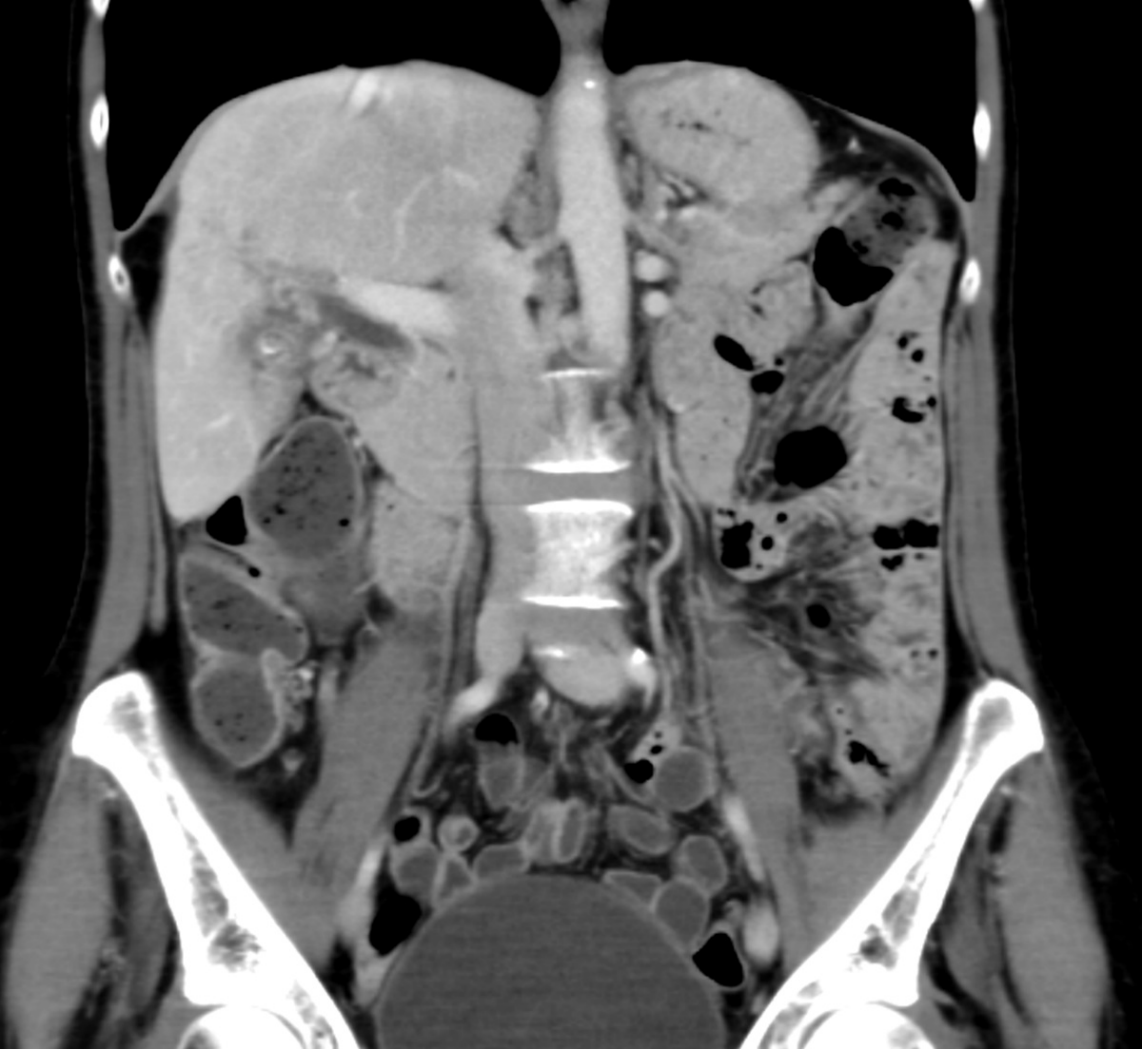
Representative coronal slice from CT obtained nine months after second SIRT procedure

Clinical follow-up, biological marker measurements, and CT scans at three, six, and nine months after her second SIRT treatment showed a dramatic reduction in tumor size and near-complete remission of disease. A three-month post-treatment CT scan revealed significant tumor response (Figures 5-6). A correlating reduction of her serum AFP from 9,260 ng/ml, at the time of diagnosis, to 6,336 ng/ml was also noted. A CT scan six months after her second SIRT procedure revealed continued response of the tumor masses in the right hepatic lobe (Figures 7-8). The reduction in the patient’s serum AFP was more dramatic from 6,336 ng/ml to 36.8 ng/ml. At the nine-month follow-up, a CT scan demonstrated near complete response of the tumor masses with a few scattered, residual, subcentimeter lesions (Figures 9-10). The patient’s serum AFP nadired into the proximity of normal ranges, at 8.3 ng/ml. Sixteen months after her treatment, the patient exhibited sustained radiographic and biochemical response to SIRT treatment of her HCC. Furthermore, she had no significant toxicities from the re-treatment procedure.

## Discussion

SIRT treatment of HCC commonly involves a single intra-arterial administration of microspheres coated in ^90^Y, a radioisotope that emits high-energy beta radiation with a half-life of ~64 hours and a mean tissue range of 2.5 mm. In the setting of HCC, the tumor mainly develops blood supply from the hepatic artery, in contrast with normal hepatic parenchyma, which relies predominantly on the portal vein. Because the ^90^Y-microspheres are administered via the hepatic artery, the tumor’s vascular bed receives a relatively higher burden of the radioactive sources as compared with normal hepatic parenchyma, thereby enhancing the therapeutic ratio, i.e. dose delivered to the malignancy versus dose delivered to the normal surrounding liver.

Survival, efficacy, and safety of ^90^Y-microspheres for the treatment of HCC are well-described. In 1998, Lau, et al. reported the safety of treating 71 HCC patients with ^90^Y-microspheres with a median activity of 81.1 mCi [4]. In a follow-up study, Lau, et al. reported on 81 HCC patients treated with ^90^Y-microspheres. They noted that prolonged survival was associated with lower pretreatment AFP level and a higher tumor-to-normal uptake [5]. Repeated treatments given to residual and recurrent tumors showed added benefits to survival and palliation [5]. Despite this and other retrospective reports demonstrating the feasibility of re-treatment with SIRT, there is some suggestion from studies in patients re-treated with SIRT for liver metastases that increased rates of hepatic toxicity, particularly radioembolization-induced liver damage (REILD). Furthermore, there is no consensus on the timing and indications for re-treatment, and typically, retreatment is reserved for patients with progression after initial therapy. Our patient demonstrated a partial response to the initial treatment, but due to the lack of significant response and her unwillingness to pursue systemic therapy, repeat SIRT was attempted. Moreover, Zarva, et al. reported in a small retrospective series that repeated lobar treatment, as opposed to whole liver treatments, may be better tolerated in well-selected patients, i.e. patients with adequate hepatic reserve, justifying such an aggressive approach in patients, like JA, who have a predominantly unilobar disease [6]. 

Another potential justification for repeat treatment is that despite demonstrated benefits in symptomatic relief and survival, overall radiographic response to treatment has shown significant room for improvement. The original Lau, et al. study showed 26.7% of the patients had 50% reduction in tumor size and an 89% response rate in AFP reduction [4]. The optimal method of response evaluation is still a topic of significant debate in the literature, but Iñarrairaegui, et al. showed that patients who were successfully downstaged, i.e. who had dramatic responses, and received definitive surgical treatment after SIRT, derived a clinically significant improvement in survival [2]. Multiple groups have proposed a variety of criteria to evaluate treatment response; each system with its own advantages and shortcomings. In 2006, Sangro, et al. reported radiographic response data on 24 patients with HCC treated with ^­^SIRT using response evaluation criteria in solid tumors (RECIST) criteria [7]. Tumor response was reported in 23.8% of the patients [7]. Other studies have reported RECIST-based partial responses ranging from 37-42% [2-3]. Another group, Kooby, et al. defined a partial response as a 30% reduction in the tumor’s largest diameter and, based on that definition, reported that three (11%) of the 27 patients treated with ^90^Y- microsphere-based radioembolization attained a partial response [8]. Despite the variety of definitions, complete and near complete radiographic response by any criteria is very uncommon in the SIRT literature. Hilgard, et al. reported a complete response rate of only 3% [3]. Besides the lack of a consistent definition, heterogeneous distribution of dose delivery is responsible, in large part, for such a wide range of tumor responses. Further work in post hoc dosimetric evaluation is ongoing and may help elucidate a more accurate dose-response relationship. 

## Conclusions

Reliable evaluations of tumor response are difficult to obtain. Currently, radiographic assessments guided by WHO and RECIST criteria are commonly used for an imaging response of tumor on CT and MRI scans. These techniques are limited by the difficulty in differentiating tumor from surrounding parenchyma. These techniques also do not reflect on the metabolism of the tumor. Lastly, due to the qualitative nature of response evaluation, they are susceptible to inconsistent interpretation across multiple examiners.  From recent non-randomized series, the overall tumor response rate to SIRT has been about 30%, but complete or near complete responses are <5%. It is even rarer for patients with a large HCC to achieve a complete response after SIRT. After two radioembolization treatments, our patient, JA, achieved a near-complete tumor response on clinical, radiographic, and biochemical assessment.
